# A Review of Psychiatric Comorbidity in Myasthenia Gravis

**DOI:** 10.7759/cureus.9184

**Published:** 2020-07-14

**Authors:** Christina Law, Claire V Flaherty, Sankar Bandyopadhyay

**Affiliations:** 1 Medicine, Penn State College of Medicine, Penn State Health Milton S. Hershey Medical Center, Hershey, USA; 2 Neurology, Penn State Health Milton S. Hershey Medical Center, Hershey, USA

**Keywords:** myasthenia gravis, depression, anxiety, mood disorders, myasthenic crisis, health related quality of life

## Abstract

This study aimed to review studies focused on the affective comorbidities associated with myasthenia gravis and to determine the extent to which neuromuscular treatment modalities address non-somatic aspects of autoimmune myasthenia gravis. Depression, anxiety, and emotional hyperactivity can aggravate myasthenia gravis, hinder accurate diagnoses, and presumably influence overall health-related quality of life.

Studies were identified using PubMed Medline and Web of Science to assess the effects of psychological factors on myasthenia gravis, encompassing 49 years of research worldwide. After analysis, approximately 6,060 patients from 32 studies worldwide between 1971 and 2020 were included. Standard-of-care approaches to diagnosis and treatment continue to under-appreciate the prevalence or impact of mood disorders in myasthenia gravis.

The majority of studies evaluated demonstrated an association between myasthenia gravis and mood disorders. However, the initiative to detect and treat affective comorbidities probably remains suboptimal. Although treatments for the somatic effects of myasthenia gravis have evolved over the past century, the paradigm of clinical practice has yet to adequately address the management of psychological impacts on the disease. This review is hoped to raise the necessary awareness in this regard.

## Introduction and background

Although generalized myasthenia gravis (MG) and the psychosocial factors associated with it have been well studied, the latter continue to be inadequately addressed clinically. MG is characterized by chronic, fatigable muscle weakness induced by autoantibodies to the Acetylcholine receptors of the neuromuscular junction. Bulbar, ocular, and respiratory muscle involvement can result in ptosis, diplopia, dysarthria, dysphagia, and respiratory failure. Despite almost a century of progress in the development of effective treatments for the somatic symptoms of MG, little is known about the exact relationship between MG and psychological disorders that often accompany it.

Occurring in 41% of MG patients, mood disorders are the most common comorbidity in neurological conditions [1]. In particular, anxiety and depression are often misdiagnosed and under-treated [2,3]. Mood changes, fatigue, shortness of breath, social withdrawal, anxiety, and depression occur in both MG and primary psychiatric conditions, which may lead to misdiagnosis and improper or delayed treatments [2,3]. Deducing the etiology of psychological symptoms is essential for not only determining the appropriate treatment but also preventing worsening of MG, as heightened anxiety can aggravate the clinical course of MG. In this systematic review analyzing 49 years of research, we attempt to elucidate the relationship between MG and mood disorders in order to provide clinically relevant guidelines for managing MG in the presence of psychological comorbidities.

## Review

 Methodology

**Figure 1:**
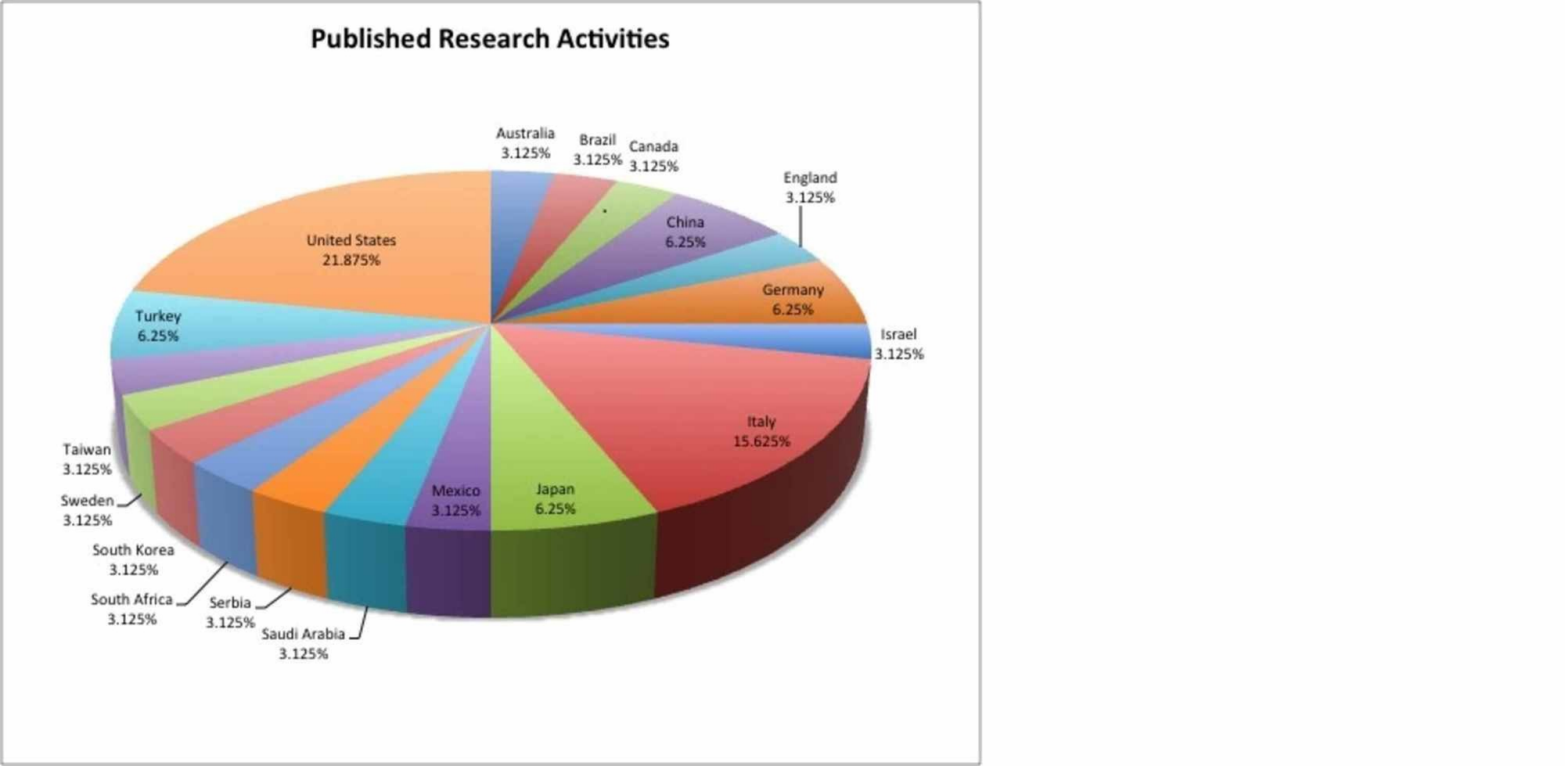
Published research activities (distribution of published work in 32 countries from the year 1971 to 2020). distribution of published work in 32 countries from the year 1971 to 2020.

**Figure 2:**
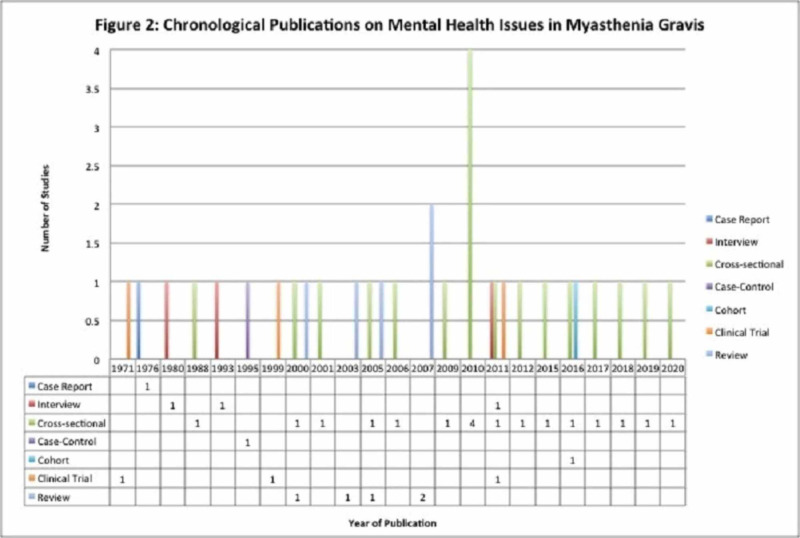
Chronological publications on mental health issues in myasthenia gravis.

A literature search for the terms “myasthenia gravis AND mood disorders”, “myasthenia gravis AND depression”, “myasthenia gravis AND anxiety”, “myasthenia gravis AND psychological stress”, “myasthenia gravis AND emotional stress”, “myasthenia gravis AND mental health”, “myasthenia gravis AND psychology”, “myasthenia gravis AND quality of life”, “myasthenia gravis AND mood disorders”, and “myasthenia gravis AND epidemiology” was conducted on PubMed MEDLINE databases for articles published between 1971 and 2020 worldwide. An additional search was made on Web of Science. Inclusion criteria were full-length articles published or available in English language on human subjects, time base of 1971 to 2020, and articles focusing on the psychological impact of autoimmune MG only and not other autoimmune or neuromuscular disorders. The exclusion criterion was inclusion of other autoimmune conditions or neuro-muscular conditions along with MG. Articles with or without quantitative data were considered. Those with quantitative data were used for quantitative analysis, whereas those without quantitative data were used to study the extent of the relationship between psychiatric comorbidity and MG across the continents over the last five decades. A total of 32 two peer-reviewed publications from six continents spanning across Australia, Brazil, Canada, China, England, Germany, Israel, Italy, Japan, Mexico, Saudi Arabia, Serbia, South Africa, South Korea, Sweden, Taiwan, Turkey, and the United States were analyzed based on study design, sample size, statistical significance, and inquiry of outcomes (Figures 1, 2). One article was excluded as per the exclusion criterion mentioned above. Overall, 6,060 patients were studied collectively. Statistical tests for significance of psychological factors on MG outcomes were assessed by the rigors of methodologies and reported p-values in each study. Qualitative studies were examined to illuminate factors that influence mood and health-related quality of life (HRQoL) in MG patients. Due to the enormous heterogeneity and differences in standards of research publications spanning over five decades, the components of Preferred Reporting Items for Systematic Reviews and Meta-Analyses (PRISMA) could not be always fulfilled. Records were identified through database searching and an additional source, records with duplication were excluded, and full-text articles were assessed and included as per the criteria set; some studies were used for quantitative analysis, whereas others were valued for their contributions toward qualitatively establishing the link between psychiatric diseases and MG.

Results

Depression

While the lifetime prevalence of major depression among the general population ranges from 7% to 17% with a 30-day prevalence of 5%, patients with a chronic illness have a 54% higher lifetime prevalence of depression with a 62% increase in six-month prevalence compared with their healthy counterparts [4-6]. Magni et al. found that a third of individuals with MG suffered from depression, which is consistent with the findings from Twork et al. [7-9]. A cross-sectional study in Saudi Arabia reported that the odds of depression were twofold higher in MG patients compared with individuals in primary care clinics (OR = 2.05; 95% CI = 1.30-3.11; p = 0.002) [10]. Longer MG disease duration, disease severity, and MG-induced respiratory failure were associated with increased rates of depression among MG patients [11,12]. In contrast, Paul et al. failed to unearth a statistically significant difference in the prevalence of depression between healthy controls and those afflicted with MG [12]. However, their study was limited by a small sample size of 29 patients [12].

Depression has been found to be a statistically significant prognostic indicator of poor HRQoL in MG patients by linear regression analysis in studies of patient samples as far reaching as Europe and Australia (p < 0.01) [13-15]. Depression is associated with decreased motivation for self-care and significant limitations in physical abilities along with increased mortality rates [9,16,17]. Likewise, depression also correlates with increased somatic complaints, ambulatory visits, and healthcare costs, all of which may contribute to poor HRQoL in MG patients [18-20].

Anxiety

Similar to the high rates of depression in debilitating diseases, MG patients have been diagnosed with an anxiety disorder in up to 46.3% of patients [12]. According to a study by Basta et al., utilizing the Hamilton Anxiety Rating Scale and multivariate linear regression analysis, anxiety is a statistically significant (β= -1.914, 95% CI: -2.44 to -1.39, p = 0.001) predictor of poor HRQoL in individuals with MG [13]. Since MG is a chronic and unpredictable illness in which repetitive use results in muscle fatigue and weakness, and physical and emotional stressors may induce myasthenic exacerbations, patients with MG often develop behavioral patterns of excessive advanced planning and avoidance of social situations to preserve muscle strength and evade embarrassment [9]. In particular, bulbar dysfunction resulting in dysarthria, dysphagia, and dysphonia was associated with statistically significant poor HRQoL (p = 0.01), as patients developed social anxiety from impaired verbal and non-verbal communication due to compromised speech, communication, facial expression, and swallowing abilities [9,11,14,15,21-23]. Restriction of social interactions may be associated with lower satisfaction with life [24]. Patients with bulbar deficiency who advanced to ventilator support had a poorer HRQoL prior to ventilation from both the physical demands of increased work of breathing and the emotional distress of anticipatory planning to circumvent social anxiety [15].

Not surprisingly, patients with respiratory failure as a direct result of MG experience greater anxiety than those without respiratory symptoms [12]. Experts from leading studies have postulated that the higher rates of anxiety disorders, including generalized anxiety, panic disorder, and agoraphobia, are attributed to the unpredictable, fluctuating nature of respiratory dysfunction, which can be exacerbated by physical or emotional stress [12,25]. Higher rates of anxiety in MG patients with respiratory distress are thus attributed to an element of anticipatory anxiety, given the presence of erratic, potentially life-threatening episodes of significant respiratory distress and apprehension about treatment with mechanical ventilation [12].

Effects of MG Treatment on Mood

One method of rapidly ameliorating weakness during a myasthenic crisis is to treat patients with plasmapheresis. Researchers have postulated that affective disturbances in MG may arise in response to the physical limitations imposed by muscle weakness and consequently may be reduced by alleviating somatic symptoms [14,25,26]. However, a study by Chen et al. revealed that while plasmapheresis effectively cleared 56% of the pathogenic antibodies, correlating with a 46% reduction in MG score, as an indicator of decreased weakness, plasmapheresis failed to reduce rates of depression and anxiety [26]. Furthermore, Mean Mental Components of HRQoL remained low post-plasmapheresis, despite improved physical function and strength [26]. While plasmapheresis reduced weakness and alleviated physical disability; it had no significant impact on diminishing mood disorders or improving mental HRQoL, suggesting that depression and anxiety may represent emotional responses to the progression of MG associated physical disabilities in the chronic course, that remain intractable throughout the early stage of the physical recovery process [26]. Consistent with this, the retrospective CONSORT study of 541 MG cases in China found a statistically significant, independent association between postoperative myasthenic crises following thymectomies and preoperative anxiety by multivariate linear regression analysis (OR = 2.40; 95% CI: 1.33-4.34; p = 0.004) [27].

In contrast to plasmapheresis, corticosteroids, first-line agents used to alleviate weakness by suppressing the production of pathogenic autoantibodies, can provoke psychiatric morbidity. This further complicates the relationship between MG and mood disorders. Suzuki et al. found that a mean daily dose of 8.1 mg of prednisolone was the most significant independent factor associated with depression in MG by multivariate linear regression analysis (OR: 1.09; 95% CI 1.02-1.17; p =0.01) [28,29]. Long-term corticosteroid use in MG has also been linked to depression, anxiety, and psychosis in two large meta-analyses [30,31]. Several studies have independently concluded that higher doses of corticosteroids precipitated depressive states in MG, with psychiatric comorbidities emerging at 10 mg/day of prednisone or prednisolone [32]. Steroids can also cause insomnia, which is an essential feature of depression and anxiety, further obscuring the etiology of mood disorders in MG treated with corticosteroids [33].

Additional Factors Affecting Psychiatric Comorbidities

Lack of social support, poor coping mechanisms and acceptance of disease, older age of onset, lower education, occupations associated with increased physical labor, career changes associated with MG, and worsening disease severity are independently statistically significant for associations with diminished HRQoL, which, in turn, can induce emotional stress in individuals afflicted with MG [14,15,27,34-37]. In a cyclical fashion, emotional turmoil can further trigger myasthenic exacerbations, resulting in debilitating physical symptoms [15,38]. Conversely, higher education and intellectual occupations requiring little physical exertion were associated with enhanced HRQoL by multivariate linear regression [14]. With fewer work restrictions, patients experienced a greater sense of satisfaction and purpose in life along with a steady income, which may also contribute to an uplifted mood and an enriched quality of life overall [14].

Effects of Psychoactive Medicines on MG

Limited observational studies have validated the potential utility of fluoxetine and psychotherapy to manage psychiatric dysfunction in patients with MG, without serious adverse effects [39,40]. In contrast, the tricyclic antidepressant amitriptyline and antipsychotic agent haloperidol aggravate MG by impairing transmission at neuromuscular junctions [41]. Diazepam has also been reported to provoke respiratory stress in MG, whereas lithium carbonate has been known to induce new-onset MG [42,43]. Effective knowledge in this regard is essential in determining a first-line treatment for managing depression and anxiety in individuals afflicted with MG.

 *Public Health Cost Burden*

**Figure 3:**
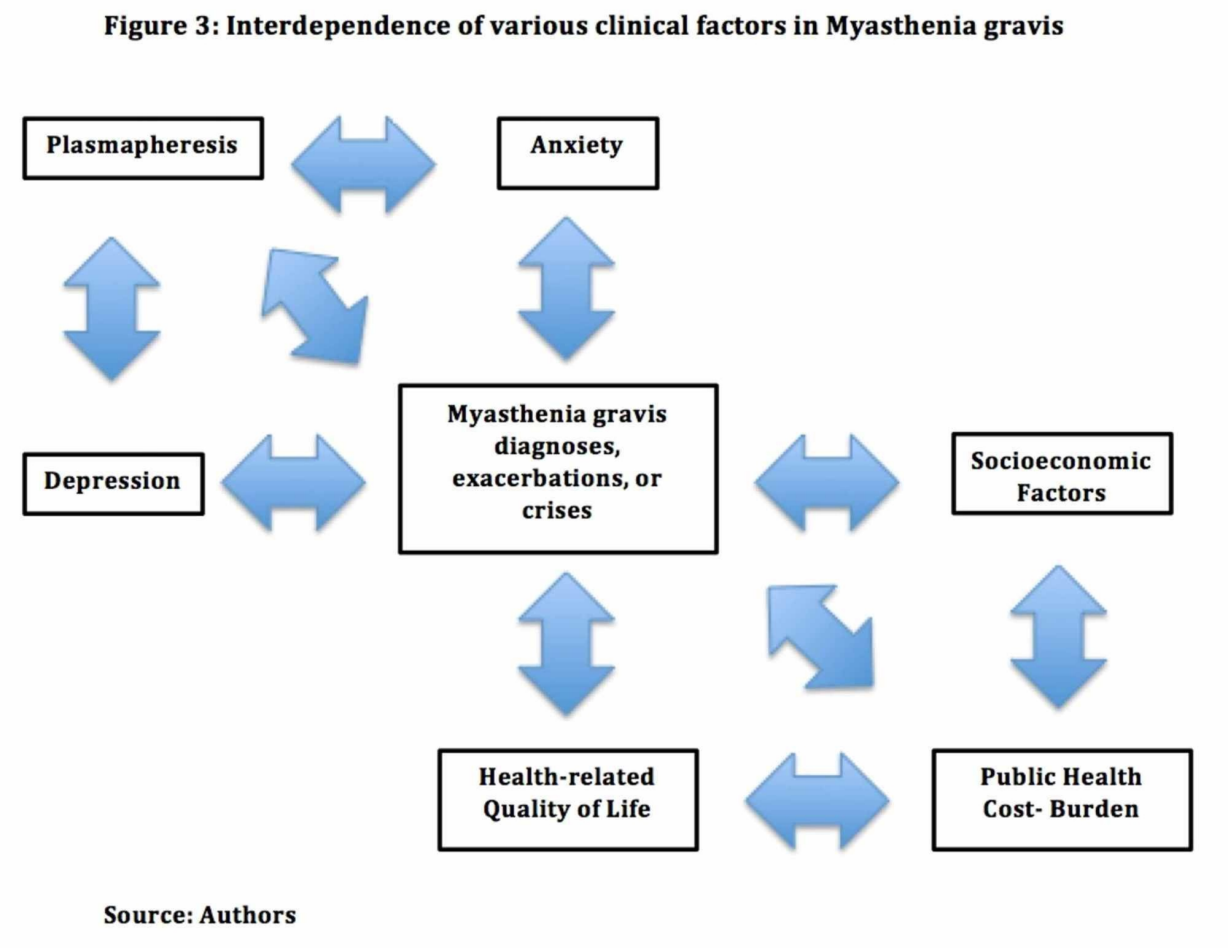
Interdependence of various clinical factors in myasthenia gravis.

The average interval between disease onset in MG and remission is four years [44]. Most patients experience one of more exacerbation of symptoms at that time. As a chronic illness with long-term disability, MG has a major impact on public health (Figure 3) [9]. In a study by Blum et al., only 40.6% of Australian individuals diagnosed with MG were able to continue working, whereas 57.6% were forced to take sick leave and 52.7% relied on government support [14]. Similarly, a recent cross-sectional multicenter study reported that 4.1% of Japanese patients with MG were unwillingly transferred, 47.1% had a reduction in 50% or more of their total income, and 27.2% were unemployed; with 49% of individuals experiencing reduced positivity associated with the social and psychological disadvantages of MG [44]. Schepelmann et al. calculated that the total annual cost of MG treatment in Germany in 2010 was €14,950 (95% CI: 10,740-21,730) per patient [45]. Guptill et al. estimated the annual cost of MG treatment in the United States in 2011 from available data on 1,288 patients diagnosed with MG [46,47]. The total annual MG-related pharmacy costs were $9.4 million, with costs related to the treatment of MG higher than those of many other chronic neurological diseases. On an individual level, occupational loss not only results in deteriorating finances and diminished access to healthcare but also adversely impacts self-esteem and sense of self-worth, representing yet another emotional stressor impeding the disease recovery course.

## Conclusions

Published data over nearly half of a century have demonstrated an association between MG and mood disorders. However, the initiative to detect and treat affective comorbidities remains, at the best, sub-optimal. Although treatments for the somatic effects of MG have evolved appreciably over the past century, the paradigm of clinical practice has yet to adequately address psychological aspects of management. Further complicating this, MG treatment with corticosteroids may induce organic affective syndromes, compounding the effects of psychological depression and anxiety, leading to poor health outcomes and a diminished quality of life. Future randomized controlled trials, while challenging to conduct, may be effective in elucidating any causal relationship between MG and psychological disorders. Whether the very autoimmune process such as the acetylcholine receptor antibodies has any direct action on the central nervous system, leading to psychological maladies, may be an interesting proposition to be pursued scientifically. As a chronic illness with long-term disability, MG has a major impact on public health on both an individual and a population level. Over the past 70 years, the mortality rate for MG has declined precipitously, currently 0.06 to 0.89 deaths per million person-years in the developed world. Improvements in intensive respiratory care and the introduction of immunosuppressive medicines have transformed this once fatal condition into a multifaceted chronic disease, extending beyond the neuromuscular junction to the emotional realm. A shift in paradigm in clinical practice is imperative to incorporate a multidisciplinary approach that addresses both the somatic and affective sequelae throughout the MG disease course. The purpose of this review article, which does not discover something altogether unknown, will be served if adequate attention leading to a higher level of awareness becomes the standard of medical care in this regard.

## References

[1] Kanner AM (2005). Should neurologists be trained to recognize and treat co-morbid depression of neurological disorders? Yes. Epilepsy Behav.

[2] Kulaksizoglu IB, Gurvit H, Polat A (2005). Unrecognized depression in community-dwelling elderly persons in Istanbul. Int Psychogeriatr.

[3] Kanner AM, Barry JJ (2003). The impact of mood disorders in neurological diseases: should neurologists be concerned?. Epilepsy Behav.

[4] Blazer DG, Kessler RC, McGonagle KA, Swartz MS (1994). The prevalence and distribution of major depression in a national community sample: the National Comorbidity Survey. Am J Psychiatry.

[5] Kessler RC, McGonagle KA, Zhao S (1994). Lifetime and 12-month prevalence of DSM-III-R psychiatric disorders in the United States. Arch Gen Psychiatry.

[6] Wells KB, Golding JM, Burnam MA (1988). Psychiatric disorder in a sample of the general population with and without chronic medical conditions. Am J Psychiatry.

[7] Magni G, Micaglio GF, Lalli R, Bejato L, Candeago MR, Merskey H, Angelini C (1988). Psychiatric disturbances associated with myasthenia gravis. Acta Psychiatr Scand.

[8] Twork S, Wiesmeth S, Klewer J, Pohlau D, Kugler J (2010). Quality of life and life circumstances in German myasthenia gravis patients. Health Qual Life Outcomes.

[9] Alanazy MH (2019). Prevalence and associated factors of depressive symptoms in patients with myasthenia gravis: a cross-sectional study of two tertiary hospitals in Riyadh, Saudi Arabia. Behav Neurol.

[10] Jeong A, Min JH, Kang YK, Kim J, Choi M, Seok JM, Kim BJ (2018). Factors associated with quality of life of people with myasthenia gravis. PLoS One.

[11] Ybarra MI, Kummer A, Frota ER, Oliveira JT, Gomez RS, Teixeira AL (2011). Psychiatric disorders in myasthenia gravis. Arq Neuropsiquiatr.

[12] Paul RH, Cohen RA, Goldstein JM, Gilchrist JM (2000). Severity of mood, self-explorative and vegetative symptoms of depression in myasthenia gravis. J Neuropsychiatry Clin Neurosci.

[13] Basta IZ, Pekmezovic TD, Peric SZ, Kisić-Tepavčević DB, Rakočević-Stojanović VM, Stević ZD, Lavrnić DV (2012). Assessment of health-related quality of life in patients with myasthenia gravis in Belgrade (Serbia). Neurol Sci.

[14] Blum S, Lee D, Gillis D, McEniery DF, Reddel S, McCombe P (2015). Clinical features and impact of myasthenia gravis disease in Australian patients. J Clin Neurosci.

[15] Carney RM, Freedland KE, Eisen SA, Rich MW, Jaffe AS (1995). Major depression and medication adherence in elderly patients with coronary artery disease. Health Psychol.

[16] Wai L, Richmond J, Burton H, Lindsay RM (1981). Influence of psychosocial factors on survival of home-dialysis patients. Lancet.

[17] Katon W, Ciechanowski P (2002). The impact of major depression on chronic medical illness. J Psychosom Res.

[18] Regier DA, Hirschfeld RM, Goodwin FK, Burke JD Jr, Lazar JB, Judd LL (1988). The NIMH depression awareness, recognition and treatment program structure, aims, and scientific basis. Am J Psychiatry.

[19] Simon GE, VonKorff M, Barlow W (1995). Health care costs of primary care patients with recognized depression. Arch Gen Pscyhiatry.

[20] Bartel PR, Lotz BP (1995). Neuropsychological test performance and affect in myasthenia gravis. Acta Neurol Scand.

[21] Padua L, Evoli A, Aprile I (2001). Health-related quality of life in patients with myasthenia gravis and the relationship between patient-oriented assessment and conventional measurements. Neurol Sci.

[22] Rostedt A, Padua L, Stalberg EV (2006). Correlation between regional myasthenic weakness and mental aspects of quality of life. Eur J Neurol.

[23] Paradis CM, Friedman S, Lazar RM, Kula RW (1993). Anxiety disorders in a neuromuscular clinic. Am J Psychiatry.

[24] Raggi A, Leonardi M, Antozzi C, Confalonieri P, Maggi L, Cornelio F, Mantegazza R (2010). Concordance between severity of disease, disability and health-related quality of life in myasthenia gravis. Neurol Sci.

[25] Kulaksizoglu IB (2007). Mood and anxiety disorders in patients with myasthenia gravis: aetiology, diagnosis and treatment. CNS Drugs.

[26] Chen YT, Chang Y, Chiu HC, Yeh JH (2011). Psychosocial aspects in myasthenic patients treated by plasmapheresis. J Neurol.

[27] Zou J, Su C, Lun X (2016). Preoperative anxiety in patients with myasthenia gravis and risk for myasthenic crisis after extended transsternal thymectomy: a CONSORT study. Medicine (Baltimore).

[28] Suzuki Y, Utsugisawa K, Suzuki S (2011). Factors associated with depressive state in patients with myasthenia gravis: a multicenter cross-sectional study. BMJ Open.

[29] Schmidt LA, Fox NA, Goldberg MC, Smith CC, Schulkin J (1999). Effects of acute prednisone administration on memory, attention, and emotion in healthy human adults. Psychoneuroimmunol.

[30] Pretorius E (2004). Corticosteroids, depression and the role of serotonin. Rev Neurosci.

[31] Warrington TP, Botswick JM (2006). Psychiatric adverse effects of corticosteroids. Mayo Clin Proc.

[32] Garcia-Carrasco M, Escarcega RO, Fuentes-Alexandro S, Riebeling C, Cercera R (2007). Therapeutic options in autoimmune myasthenia gravis. Autoimmune Rev.

[33] Leonardi M, Raggi A, Antozzi C, Confalonieri P, Maggi L, Cornelio F, Mantegazza R (2010). The relationship between health, disability and quality of life in myasthenia gravis: results from an Italian study. J Neurol.

[34] Raggi A, Leonardi M, Mantegazza R, Casale S, Fioravanti G (2009). Social support and self-efficacy in patients with myasthenia gravis: a common pathway towards positive health outcomes. Neurol Sci.

[35] Yang Y, Zhang M, Guo J (2016). Quality of life in 188 patients with myasthenia gravis in China. Int J Neurosci.

[36] Bogdan A, Barnett C, Ali A, AlQwaifly M, Abraham A, Mannan S, Ng E, Bril V (2020). Chronic stress, depression and personality type in patients with myasthenia gravis. Eur J Neurol.

[37] Sneddon J (1980). Myasthenia gravis: a study of social, medical and emotional problems in 26 patients. Lancet.

[38] Achiron A, Barak Y, Noy S, Pinhas-Hamiel O (1999). Fluoxetine treatment for weight reduction in steroid-induced obesity: a pilot study in myasthenia gravis patients. Eur Neuropsychopharmacol.

[39] Schwartz ML, Cahill R (1971). Psychopathology associated with myasthenia gravis and its treatment by psychotherapeutically oriented group counseling. J Chron Dis.

[40] Argov Z, Mastaglia FL (1979). Disorders of neuromuscular transmission caused by drugs. N Engl J Med.

[41] Kramer M (2000). Hypnotic medication in the treatment of chronic insomnia: non nocere! Doesn’t anyone care?. Sleep Med Rev.

[42] Neil JF, Himmelhoch JM, Licata SM (1976). Emergence of myasthenia gravis during treatment with lithium carbonate. Arch Gen Psychiatry.

[43] Hehir Hehir, MK MK, Silvestri NJ (2018). Generalized myasthenia gravis: classification, clinical presentation, natural history, and epidemiology. Neurol Clin.

[44] Nagane Y, Murai H, Imai T (2017). Social disadvantages associated with myasthenia gravis and its treatment: a multicentre cross-sectional study. BMJ Open.

[45] Schepelmann K, Winter Y, Spottke AE (2010). Socioeconomic burden of Amytrophic lateral sclerosis, myasthenia gravis and fascioscapulohumeral muscular dystrophy. J Neurol.

[46] Guptill. JT, Marano A, Krueger A, Sanders DB (2011). Cost analysis of myasthenia gravis from a large U.S. insurance database. Muscle Nerve.

[47] Carr AS, Cardwell CR, McCarron PO, McConville J (2010). A systematic review of population based epidemiological studies in myasthenia gravis. BMC Neurol.

